# Transactions of the Louisiana State Medical Society, 1884

**Published:** 1884-12

**Authors:** 


TRANSACTIONS OF THE LOUISIANA STATE MEDICAL SOCIETY at its sixth annual session, held at Baton Rouge, La., May 21, 22 and 23, 1884, New Orleans. L. Graham & Son, Printers, 137 Gravier street.

A paper-covered pamphlet of one hundred pages with the above title page, accompanied by a printed slip of paper, inviting criticism for its contents, has been forwarded to us, with the compliments of the Louisiana State Medical Society.

We are struck at the outset of the proceedings with the failure of the Committee of Arrangements to present any report. It is with surprise we note that no reference appears to the announcement of Dr. E. S. Lewis, that he would receive subscriptions for a monument to Dr. Sims.

In a paper by Dr. G. B. Underhill on the spleen, "his conclusion was that the malarial poison was purely a nervine poison, affecting primarily the cerebro-spinal nerve fibres situated in the sympathetic tracts, and by inhibiting the action of the latter, causes a dilatation of the vessels of all the greater digestive viscera, and especially the spleen." By this it seems one step has been taken towards the elucidation of "the unknown factor in the equation of the nervous system," and more especially as it is stated that "a number of cases submitted seem to substantiate the opinions expressed in the paper."

It crops out that somebody is not entirely content with our time-honored anesthetic, as "Dr. Underhill moved the appointment of a committee to send circulars to each member of this Society, inquiring as to the extent of the use of chloroform, the mode of administration, and the number of deaths resulting therefrom," which was, upon motion of Dr. Chailli, amended, so "that the circular be sent to physicians outside of the Society as well as to the members," and thus was passed. If the proposition had included sulphuric ether, nitrite of amyl and bromide of ethyl, it would have given more satisfactory results in the reports.

At the invitation of the Society, Judge Leay stated that the first point for legislation was the establishment of a State Board of

Health, composed of representative physicians and others, from all parts of the State, with the Secretary a physician, and with powers both corporate and politic. The second recommendation was the protection of confidential communications by patients to physicians. He could see no difficulty in the passage of this bill. He was also in favor of sanitary instruction, so "that the public schools should teach elementary physiology and hygiene," without noticing that in this, as in other matters, a little learning is a dangerous thing. He favored the proposed law "checking the too frequent pleading of insanity for crime, and the exemption from punishment, and the taking care of the actually insane," and also that looking to "compensation for expert testimony" by physicians.

But the trouble lies not so much in the law as in the courts, and in the partisan evidence given by doctors on the stand, so that we must look to the proper administration of justice, and the proper qualification of a medical witness in trials involving the question of insanity.

An elaborate paper on cremation was presented and read by Dr. F. Formento, in which he urged its claims in a sanitary, social, moral, and economical point of view; stating its special adaptation to the population and surroundings of the city of New Orleans; and his views were adopted as the sense of the society by a formal vote. The example of Dr. Gross is contagious for M. Ds.

The society resolved to forward by every means in their power the plans by which the managers of the "Exposition" propose to illustrate the natural history and resources of the State.

Colonel T. C. Zacharie, the Attorney of the Board of Health, was called upon to elucidate their plans in regard to quarantine; but really made confusion worse confounded. After some remarks on the part of Dr. S. Jones and Dr. Chaille, Dr. T. J. Buffington offered the following, which was carried:

"*Resolved*, That in case the Board of Health should deem it necessary to protect the State of Louisiana against the introduction of any infectious or contagious diseases, to adopt absolute non-intercourse with infected or suspected localities, their course will meet with the approval of this Society."

So much for exclusion, that may avail little, unless rigid internal sanitation is observed.

An eminently practical paper on "Acute Plastic Iritis" was read by Dr. Henry Dickson Burns, but he lacks the crowning measure of treatment in not resorting to iodide of potash after the mercurial course.

A most admirable annual oration on "The Scientific Method" was made by Col. K. A. Cross; and it is well calculated to corrert some of the vagaries of evolutionists from reasoning in a vicious circle.

Organization of a proper kind is the one thing needful for this Society, and it is to be expected that all will go well under the presidency of Dr. Day.

